# Association of tumor necrosis factor-α gene polymorphisms and coronary artery disease susceptibility: a systematic review and meta-analysis

**DOI:** 10.1186/s12881-020-0952-2

**Published:** 2020-02-11

**Authors:** Rui Huang, Su-Rui Zhao, Ya Li, Fang Liu, Yue Gong, Jun Xing, Ze-Sheng Xu

**Affiliations:** 1 Department of Cardiology, Cangzhou Central Hospital, Cangzhou, China; 2 Department of Cardiology, Cangzhou Central Hospital, Hebei Province, No. 16 Xinhua West Road, Ganzhou, 061001 China

**Keywords:** Tumor necrosis factor-α, Gene polymorphisms, Coronary artery disease, Meta-analysis

## Abstract

**Background:**

The goal of this study was to review relevant case-control studies to determine the association of tumor necrosis factor-α (*TNF-α*) gene polymorphisms and coronary artery disease (CAD) susceptibility.

**Methods:**

Using appropriate keywords, we identified relevant studies using PubMed, Cochrane, Embase, CNKI, VANFUN, and VIP. Key pertinent sources in the literature were also reviewed, and all articles published through April 2019 were considered for inclusion. Based on eligible studies, we performed a meta-analysis of association between 308G/A, 238G/A, 857C/T, 863C/A and 1031 T/C polymorphisms in *TNF-α* and risk of CAD.

**Results:**

We found 25 studies that were consistent with this meta-analysis, including 7697 patients in the CAD group and 9655 control patients. *TNF-α* 308G/A locus A showed no significant association with CAD susceptibility by the five models in the analysis of the overall population, European, African, South Asian, and North Asian patients. *TNF-α* 863C/A locus A and 1031 T/C locus C exhibited no significant association with CAD susceptibility. *TNF-α* 238G/A locus A had no significant association with CAD susceptibility in the overall population. However, *TNF-α* 238G/A locus A showed significant association with higher CAD susceptibility in the subgroup of Europeans and north Asians. *TNF-α* 857C/T locus T had no significant association with CAD susceptibility in the analysis of the overall population and Europeans. In the north Asian population, *TNF-α* 857C/T locus T was associated with lower CAD susceptibility by the heterozygote model.

**Conclusion:**

*TNF-α* 308G/A, 857C/T, 863C/A, and 1031 T/C has no significant association with CAD susceptibility. *TNF-α* 238G/A locus A has significant association with CAD susceptibility in Europeans and north Asians, but has no significant association in the overall population. Studies with a larger sample size are required to confirm the association between *TNF-α* 238G/A and CAD susceptibility.

## Background

Coronary artery disease (CAD) refers to a heart disease caused by ischemia and hypoxia of myocardial cells following coronary artery stenosis or blockage due to coronary atherosclerosis (AS). Globally, CAD is an important cause of mortality and morbidity, with approximately 9 million deaths between 2007 and 2017 [1]. At present, the major risk factors for CAD confirmed in clinical studies include age, gender, poor diet and lifestyle habits, metabolic syndrome (including obesity or overweight, hypertension, type 1 or type 2 diabetes and dyslipidemia), smoking, drinking, psychosocial factors and genetic factors. Studies [2, 3] showed that the risk of developing CAD in an individual is modulated by an interplay between genetic and lifestyle factors. In the future, genetic testing can be expected to enable precision medicine approaches by identifying subgroups of patients at increased risk of CAD or those with a specific driving pathophysiology in whom a therapeutic or preventive approach is most useful.

Tumor necrosis factor (TNF) is a proinflammatory cytokine in vivo with extensive biological activities. Human *TNF* gene, located in the short arm of chromosome 6, is a 7 kb DNA sequence composed of *TNFA* and *TNFB*, encoding TNF-α and TNF-β, respectively, each containing 4 exons and 3 introns. At present, many scholars agree that there is an interactive feedback loop between acute or chronic inflammatory reactions, the dynamics of atherosclerotic plaques, platelet aggregation, activation of the coagulation system and lipid metabolism disorders. Inflammatory response may be an important trigger mechanism, and there are many kinds of inflammatory biomarkers in serum, including C-reactive protein, intercellular adhesion molecule, *p*-selectin, amyloid A protein, fibrinogen, *e*-selectin, pregnancy-related plasma protein-a, serum interleukin-6, and TNF-α [4–6]. Studies have shown that the presence of *TNF-α* gene polymorphism may affect gene transcription and expression levels, and is associated with a variety of diseases such as rheumatoid arthritis, type 1 diabetes, type 2 diabetes, ankylosing spondylitis, sarcoidosis, and silicosis [7–9]. The aim of this study was to perform a meta-analysis of all available literature to obtain updated evidence about association between *TNF-α* polymorphisms and CAD susceptibility.

## Methods

### Search strategy

To identify studies pertaining to the associations between 308G/A, 238G/A, 857C/T, 863C/A and 1031 T/C polymorphisms in *TNF-α* and risk of CAD, we reviewed the Cochrane library, PubMed, Embase, CNKI, VANFUN, and VIP databases for relevant articles published through April 2019. We also reviewed the references of all identified articles to look for additional studies. Search terms were as follows: gene polymorphisms, gene, polymorphism, variant, genotype, tumor necrosis factor-α, TNF-α, coronary artery disease, CAD, angina, myocardial infarction, ischemic heart disease, tumor necrosis factor and TNF. These terms were used in combination with “AND” or “OR”. This literature review was performed independently by two investigators, with a third resolving any disputes as needed. The detailed search strategy of PubMed: (“gene polymorphisms” or “gene” or “polymorphism” or “variant” or “genotype”) and (“tumor necrosis factor-α” or “TNF-α” or “tumor necrosis factor” or “TNF”) and (“coronary artery disease” or “CAD” or “angina” or “myocardial infarction” or “ischemic heart disease”) AND Humans [Mesh]Search.

Following the PICOS (Participants, Interventions, Comparisons, Outcomes and Study design) principle, the key search terms included (P) patients with CAD; (I) detection the gene polymorphisms of *TNF-α*; (C/O) compare the gene polymorphisms of *TNF-α* between the CAD group and the control group; (S) case-control studies or cohort study.

### Study selection criteria

Eligible studies met the following criteria: [1] case-control or cohort studies [2]; the subjects in the case group were patients with CAD [3]; the participants in the control group did not have CAD [4]; 308G/A, 238G/A, 857C/T, 863C/A and 1031 T/C of *TNF-α* were studied; 4) English or Chinese language.

Studies were excluded for meeting the following criteria: [1] duplicate articles or results [2]; apparen tdata errors [3]; case reports, theoretical research, conference reports, systematic reviews, meta-analyses, and other forms of research or comment not designed in a randomized controlled manner [4]; irrelevant outcomes [5]; lack of a control group.

Two investigators independently determined whether studies met the inclusion criteria, with a third resolving any disputes as needed.

### Data extraction and quality assessment

For each included study, two categories of information were extracted: basic information and primary clinical outcomes. Basic information relevant to this meta-analysis included: author names, year of publication, country, ethnicity, and sample size. Primary outcomes relevant to this analysis included frequency of genotypes (308G/A, 238G/A, 857C/T, 863C/A and1031T/C of TNF-α) in the CAD group and the control group. This data extraction was performed independently by two investigators, with a third resolving any disputes as needed.

We used Newcastle–Ottawa Scale (NOS) to assess the quality of eligible studies. The version of case-control studies included a set of questions: adequacy of case definition, representativeness of cases, selection of controls, definition of controls, matched age and sex, additional factors, ascertainment of exposure, case and controls (the same ascertainment method), cases and control (the same non-response rate).

### Statistical analysis

STATA v12.0 (TX, USA) was used for all analyses. Heterogeneity in study results was assessed using chi-squared and I^2^tests and appropriate analytic models (fixed-effects or random-effects) were determined. A chi-squared *P* ≤ 0.05 and an I^2^ > 50% indicated high heterogeneity and the random-effects model was used in this case. A chi-squared *P* > 0.05 and an I^2^ ≤ 50% indicated acceptable heterogeneity and the fixed-effects model was used. Egger’s test and Begg’s test were used to determine whether there was publication bias. Under ideal conditions (such as random mating, no selection, mutation, or migration), if the population is in line with the Hardy-Weinberg equilibrium (HWE), the proportion of certain characteristic genes will remain unchanged in inheritance. HWE is closely related to genotyping quality. HWE is a common hypothesis. In the meta-analysis of genetic association study, it is necessary to test whether the genotype distribution of the control group conforms to HWE. If the HWE genetic balance test was not provided in the original text or not performed on the control group, we used Stata v12.0 to carry out manual detection and extracted the corresponding results (*P* value). Five commonly used gene models were selected for meta-analysis: the allelic model (A vs. C); homozygote model (AA vs. CC); heterozygote model (AC vs. CC); dominant model (AA + ACvs.CC); regressive model (AA vs. AC+ CC). OR and 95% CI were used to analyze all the indexes.

## Results

### Overview of included studies

**Fig. 1:**
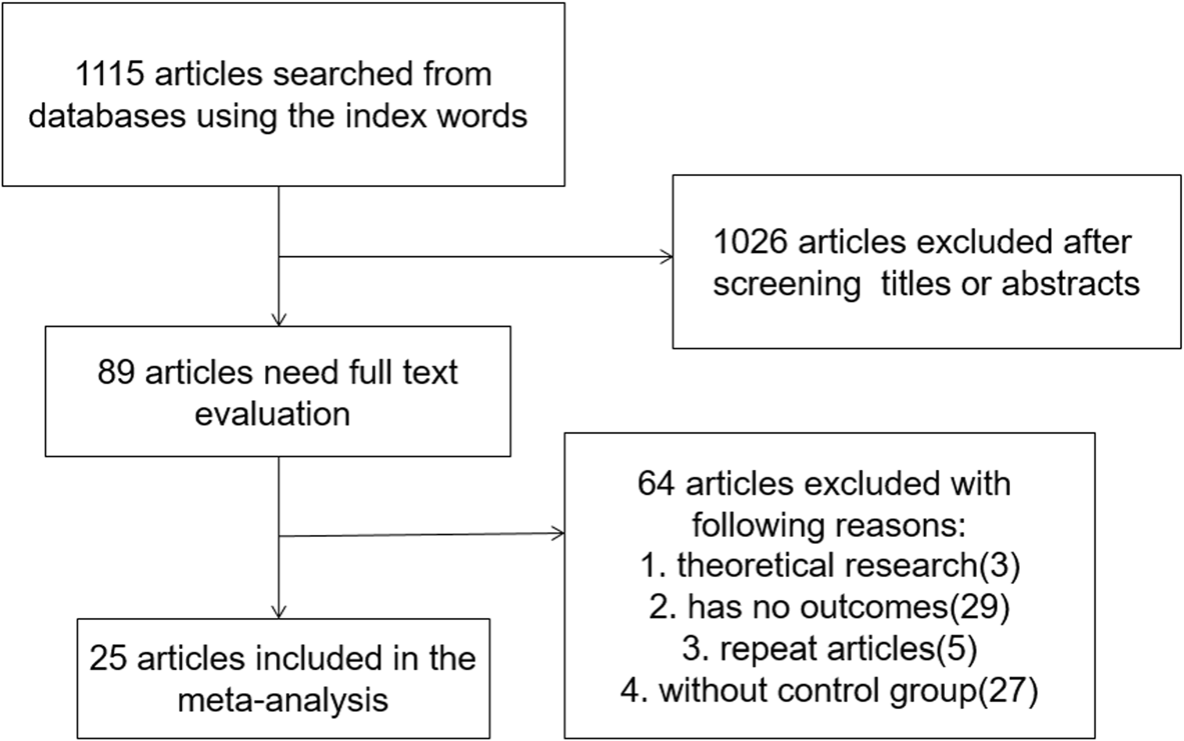
Literature search and selection strategy

**Fig. 2:**
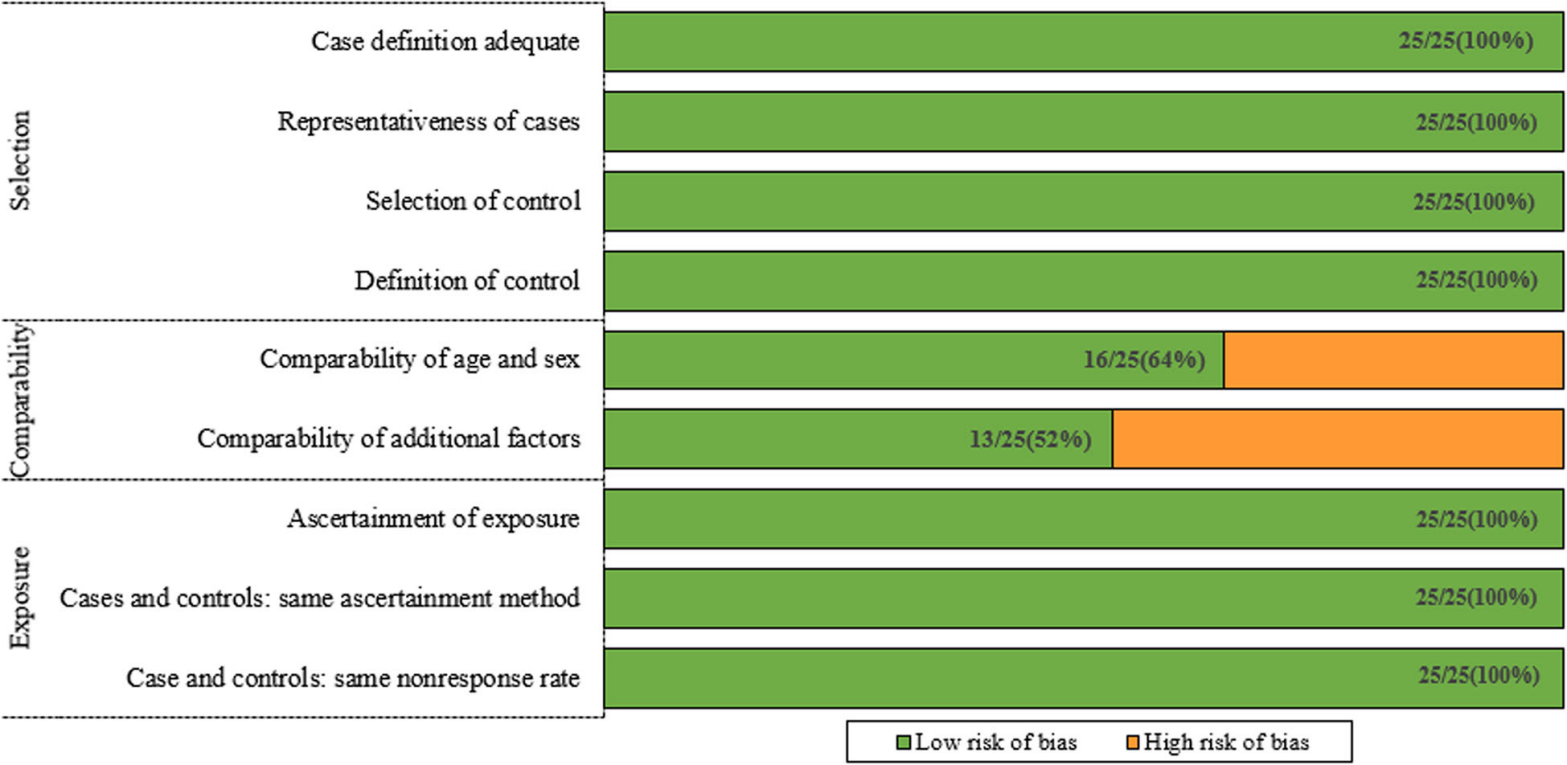
Risk of bias by domain (in bold) and question in twenty-six case-control studies using the Newcastle–Ottawa Scale

We reviewed a total of 1115 articles identified by our initial keyword search, of which 1026 were excluded following title/abstract review. The complete full texts of the remaining 89 articles were assessed, excluding 64 articles that did not meet the study inclusion criteria. Reasons for exclusion of these studies were theoretical research [3], lack of clinical outcomes [10], duplicate articles [5], and lack of a control group [11]. We ultimately identified a total of 25 case-control studies [10–34] that met the inclusion criteria for this meta-analysis, including 7697 patients in the CAD group and 9655 in the control group. The study selection process is outlined in Fig. 1. Table 1 summarizes the basic information for each study, including author names, year of publication, country, ethnicity, and sample size. Seven studies involved Eurpeans, 14 involved north Asians, 3 involved south Asians, 2 involved Africans, and 1 involved North Americans. The risk of bias assessed by NOS is presented in Fig. 2.

**Table 1:** The basic characteristics description of included studies

Study	Country	No. of patients		Age		Gender		Genetic testing method	Ethnicity
		Case group	Control group	Case group	Control group	Case group	Control group		
S. M. Herrmann et al. 1998 a	Northern Ireland	641	710	–	–	–	–	Polymerase chain reaction-single-strand conformation polymorphism	European
S. M. Herrmann et al. 1998 b	France	446	531	–	–	–	–	Polymerase chain reaction-single-strand conformation polymorphism	European
Li Yan et al. 2004	China	210	186					Polymerase chain reaction-single-strand conformation polymorphism	North Asian
A.M. Bennet et al. 2006	Sweden	1213	1561	52~67	53~68	852 M	1054 M	–	European
Liu Yan et al. 2011	China	438	330	–	–	–	–	high resolution melting	North Asian
Zhang Lei et al. 2011	China	107	115	–	–	–	–	high resolution melting	North Asian
Ho-Chan Cho et al. 2013	South Korea	197	404	61.4	62.01	130 M	263 M	–	North Asian
Qi Xiaoming et al. 2014	China	207	274	–	–	–	–	high resolution melting	North Asian
Liu Yan et al. 2009	China	286	202	–	–	–	–	matrix assisted laser desorption ionization time	North Asian
Liang Hao et al. 2011	China	121	138	–	–	84 M	92 M	Polymerase chain reaction-single-strand conformation polymorphism	North Asian
Xiang Xiaping et al. 2004	China	162	182	–	–	–	–	Enzyme - linked immunosorbent assay with double antibody sandwich	North Asian
Li Yan et al. 2003	China	112	158	–	–	–	–	–	North Asian
Sun Yujie et al. 2007	China	121	115	64.9	50.4	84 M	74 M	Polymerase chain reaction-single-strand conformation polymorphism	North Asian
Pan Min et al. 2008	China	90	115	65.6	64.06	65 M	70 M	Polymerase chain reaction-single-strand conformation polymorphism	North Asian
Zhao Xiaolei et al. 2015	China	783	749	64.82	59.74	497 M	477 M	–	North Asian
Lakhdar Ghazouani et al. 2009	Tunisia	418	406	58.1	56.7	87F	107F	–	African
Indranil Banerjee et al. 2007	India	210	232	59	56	166 M	166 M	Polymerase chain reaction-single-strand conformation polymorphism	South Asian
Elena Sandoval-Pinto et al. 2016	Mexico	251	164	65	58	187 M	71 M	Enzyme - linked immunosorbent assay with double antibody sandwich	North American
Yuting Cheng et al. 2015	China	247	304	61.13	61.31	120F	152F	–	North Asian
I. SBARSI et al. 2007	Italy	248	241	61.8	–	197 M	–	Polymerase chain reaction-single-strand conformation polymorphism	European
Robertina Giacconi et al. 2006	Italy	105	190	71.9	76	72 M	123 M	–	European
R. A. Allen et al. 2001	UK	180	250	59~63	37	117 M	124 M	–	European
P. E. Morange et al. 2008	Germany	136	1264	67	61	100 M	923 M	–	European
Liping Hou et al. 2009	China	804	905	–	–	–	–	–	North Asian
Aparna A. Bhanushali et al. 2013	India	100	150	48	50	80 M	70 M	–	South Asian
Gul Zareen Asifa et al. 2013	Pakistan	310	310	54.3	53.2	–	–	–	South Asian

F:female, M: male

### Meta-analysis of *TNF-α*308G/a polymorphisms and CAD susceptibility

In total, 19 studies with 7036 patients in the CAD group and 8940 controls reported on the association between *TNF-α* 308G/A and CAD susceptibility. For studies without significant heterogeneity (chi-squared *P* > 0.05 and I^2^ < 50%), the fixed-effects model was chosen to analyze the all the comparison models except the dominant model and allelic model in the subgroup analysis of South Asians. The results of Begg’s test (*p* > 0.05) suggested that there was no significant publication bias among the study results.

The results showed that *TNF-α* 308G/A locus A had no significant association with CAD susceptibility: the allelic model (A vs. G) (OR:1.047, 95% CI:0.973–1.126); the homozygote model (AA vs. GG) (OR:1.106,95% CI:0.888–1.377); the dominant model (AA + GA vs. GG) (OR: 1.046,95% CI:0.963–1.136); the regressive model (AA vs.GA + GG) (OR: 1.102,95% CI: 0.886–1.370); the heterozygote model (GA vs. GG) (OR: 1.037,95%CI:0.950–1.131). In the subgroup analysis, there was no significant association between TNF-α 308G/A locus A and CAD by the five models.

**Fig. 3:**
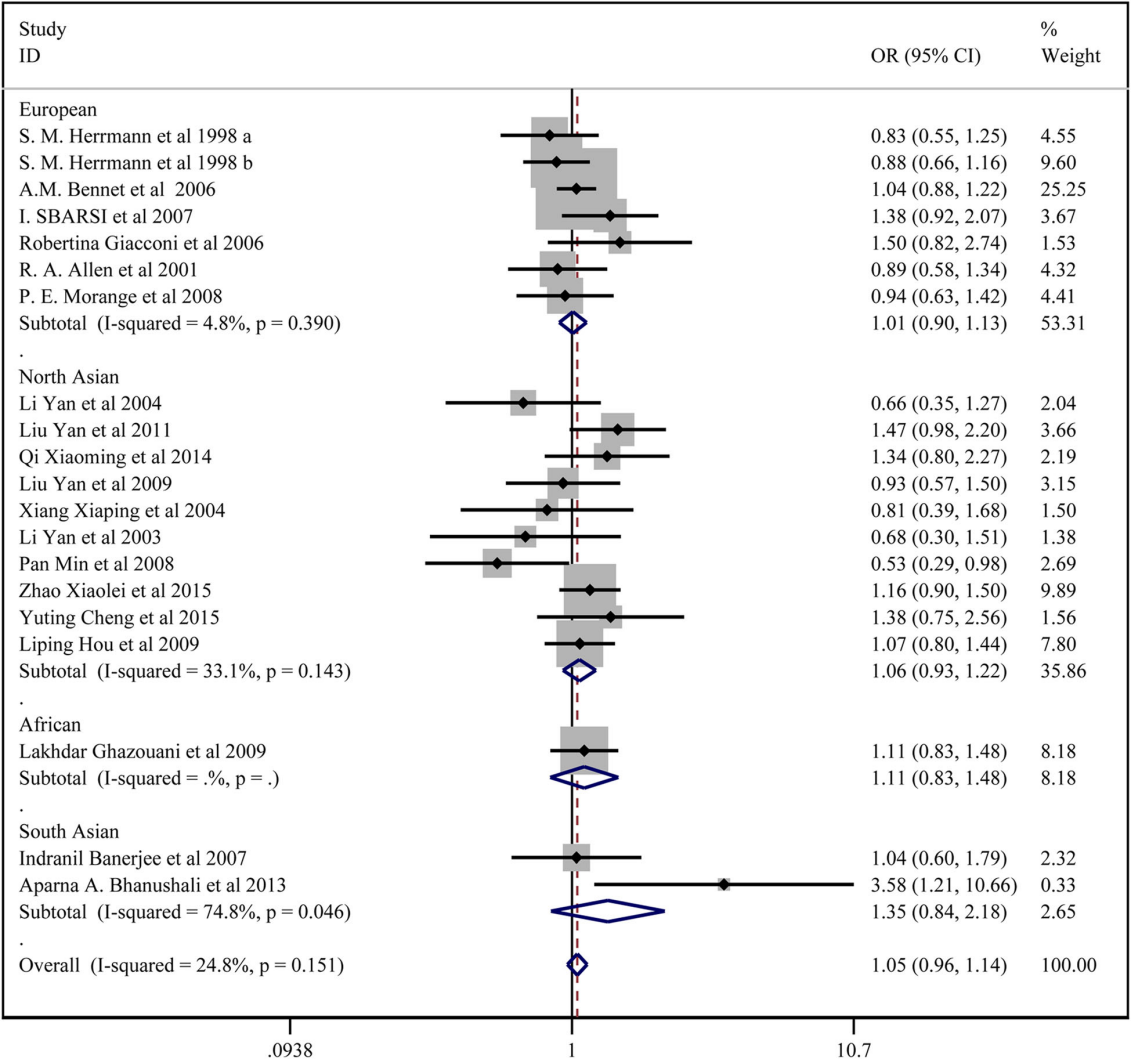
Forest plot for the dominant model of TNF-α 308G/A polymorphisms associated with CAD

**Fig. 4:**
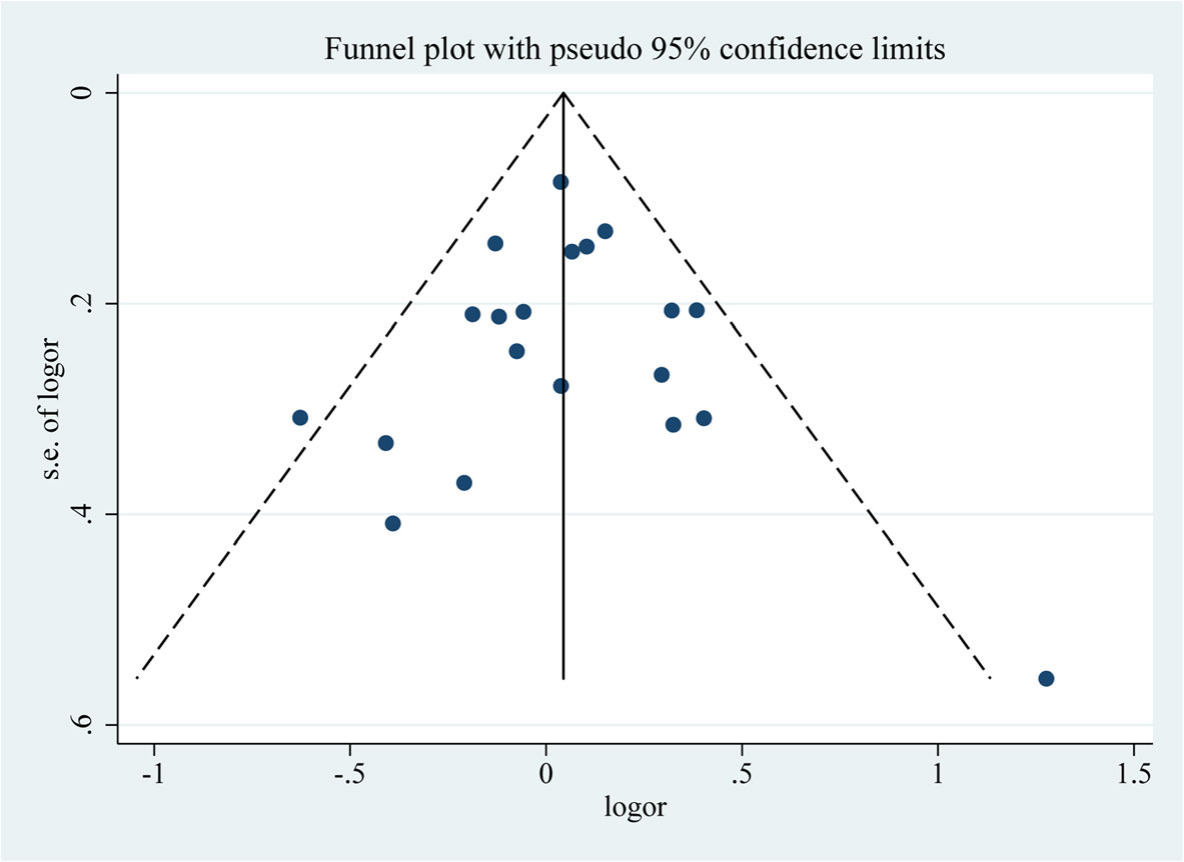
Funnel plot analysis of the included studies on*TNF-α* 308G/A polymorphisms

All the above results are presented in Fig. 3, Fig. 4 and Table 2.

**Table 2:** Meta-analysis of TNF-α 308G/A polymorphisms and CAD susceptibility

Genetic Model	Subgroup analysis	N (case/control)	OR(95% CI)	P*	I2	P#	P value	
							Begg	Egger
AA vs GG + GA								
	overall	6522/8196	1.102 (0.886,1.370)	0.209	22.0%	0.383	0.322	0.106
	European	2472/4076	1.118 (0.810,1.544)	0.087	45.7%	0.496	0.881	0.102
	North Asian	3322/3332	1.135 (0.821,1.570)	0.349	10.0%	0.443	0.624	0.907
	African	418/406	0.705 (0.320,1.553)	–	–	0.385	–	–
	South Asian	310/382	3.880 (0.401,37.525)	0.894	0.0%	0.242	0.317	–
	HWE	5814/7737	1.096 (0.835,1.438)	0.210	23.1%	0.510	0.329	0.042
	NO HWE	708/459	1.112 (0.773,1.601)	0.127	57.1%	0.567	0.317	–
AA+GA vs GG								
	overall	6522/8196	1.046(0.963,1.136)	0.151	24.8%	0.290	0.770	0.973
	European	2472/4076	1.008 (0.899,1.130)	0.390	4.8%	0.890	0.881	0.804
	North Asian	3322/3332	1.065 (0.927,1.222)	0.143	33.1%	0.374	0.128	0.138
	African	418/406	1.109 (0.833,1.476)	–	–	0.478	–	–
	South Asian	310/382	1.352 (0.839,2.179)	0.046	74.8%	0.216	0.317	–
	HWE	5814/7737	1.033 (0.948,1.126)	0.175	23.6%	0.459	0.791	0.972
	NO HWE	708/459	1.219 (0.896,1.658)	0.152	51.3%	0.208	0.317	–
AA vs GG								
	overall	6522/8196	1.106 (0.888,1.377)	0.226	20.5%	0.367	0.373	0.132
	European	2472/4076	1.105 (0.798,1.530)	0.118	41.0%	0.548	0.453	0.097
	North Asian	3322/3332	1.147 (0.829,1.587)	0.274	22.1%	0.407	0.624	0.822
	African	418/406	0.739 (0.333,1.638)	–	–	0.456	–	–
	South Asian	310/382	4.018 (0.415,38.903)	0.869	0.0%	0.230	0.317	–
	HWE	5814/7737	1.088 (0.828,1.431)	0.234	20.8%	0.545	0.329	0.048
	NO HWE	708/459	1.138 (0.790,1.641)	0.121	58.5%	0.488	0.317	–
GA vs GG								
	overall	6522/8196	1.037 (0.950,1.131)	0.258	15.7%	0.418	0.673	0.958
	European	2472/4076	0.999 (0.887,1.124)	0.352	10.1%	0.981	0.881	0.707
	North Asian	3322/3332	1.049 (0.903,1.218)	0.288	16.9%	0.531	0.531	0.398
	African	418/406	1.154 (0.859,1.549)	–	–	0.179	–	–
	South Asian	310/382	1.281 (0.790,2.076)	0.061	71.4%	0.315	0.317	–
	HWE	5814/7737	1.027 (0.940,1.122)	0.226	19.1%	0.557	0.850	0.671
	NO HWE	708/459	1.389 (0.839,2.298)	0.641	0.0%	0.201	0.317	–
A vs G								
	overall	6522/8196	1.047 (0.973,1.126)	0.065	34.7%	0.222	0.721	0.673
	European	2472/4076	1.017 (0.920,1.124)	0.303	16.6%	0.741	0.453	0.312
	North Asian	3322/3332	1.071 (0.947,1.211)	0.071	43.1%	0.276	0.128	0.120
	African	418/406	1.043 (0.816,1.332)	–	–	0.636	–	–
	South Asian	310/382	1.400 (0.887,2.210)	0.037	77.0%	0.149	0.317	–
	HWE	5814/7737	1.034 (0.957,1.116)	0.122	28.9%	0.399	0.850	0.628
	NO HWE	708/459	1.172 (0.927,1.481)	0.039	76.6%	0.185	0.317	–

*P value of Heterogeneity chi-squared

#P value of Pooled statistic

### Meta-analysis of *TNF-α* 238G/a polymorphisms and CAD susceptibility

In total, 12 studies with 5167 patients in the CAD group and 7103 controls reported on the association of *TNF-α* 238G/A and CAD susceptibility. For studies without significant heterogeneity (chi-squared *P* > 0.05 and I^2^ < 50%), the fixed-effects model was chosen to analyze the all the comparison models except the dominant model and the heterozygote model in the subgroup analysis of the overall population, north Asians and HWE, and the allelic model in the subgroup analysis of HWE. The results of Begg’s test (*p* > 0.05) suggested that there was no significant publication bias among the study results.

The results showed that *TNF-α* 238G/A locus A had no significant association with CAD susceptibility: the allelic model (A vs. G) (OR:1.088, 95% CI:0.950–1.244); the homozygote model (AA vs. GG) (OR:1.506, 95% CI:0.835–2.715); the dominant model (AA + GA vs. GG) (OR: 1.072, 95% CI:0.931–1.235); the regressive model (AA vs*.* GA + GG) (OR: 1.437, 95% CI: 0.821–2.662); the heterozygote model (GA vs GG) (OR: 1.165, 95% CI:0.914–1.485).

In the subgroup analysis, *TNF-α* 238G/A locus A showed significant association with higher CAD susceptibility in the subgroup of Europeans: the homozygote model (AA vs. GG) (OR:2.961, 95% CI:1.113–7.9879); the regressive model (AA vs. GA + GG) (OR: 2.985, 95% CI: 1.121–7.946). *TNF-α* 238G/A locus A had significant association with higher CAD susceptibility in the subgroup of HWE: the homozygote model (AA vs. GG) (OR:2.838, 95% CI:1.260–6.394); the regressive model (AA vs. GA + GG) (OR: 2.832, 95% CI: 1.258–6.375).*TNF-α* 238G/A locus A exhibited significant association with higher CAD susceptibility in the subgroup of North Asian: the dominant model (AA + GA vs. GG) (OR:1.231, 95% CI:1.010–1.500).*TNF-α* 238G/A locus A displayed significant association with higher CAD susceptibility in the subgroup of no HWE: the dominant model (AA + GA vs. GG) (OR: 1.686, 95% CI:1.060–2.681); the heterozygote model (GA vs. GG) (OR: 2.265, 95% CI:1.307–3.926).

**Fig. 5:**
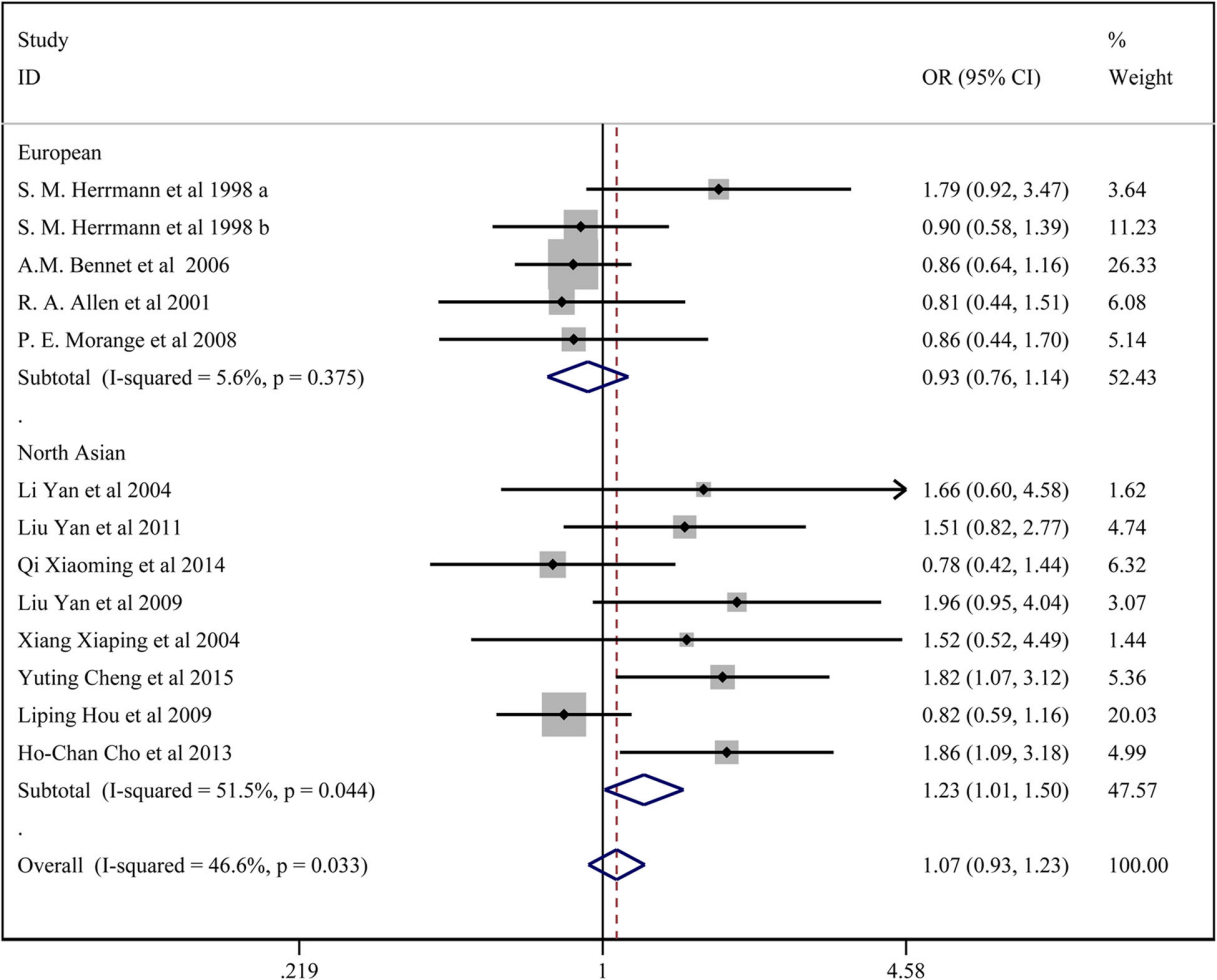
Forest plot for the dominant model of TNF-α 238G/A polymorphisms associated with CAD

All the above results are presented in Fig. 5 and Table 3.

**Table 3:** Meta-analysis of TNF-α 238G/A polymorphisms and CAD susceptibility

Genetic Model	Subgroup analysis	N (case/control)	OR(95%CI)	P*	I2	P#	P value	
							Begg	Egger
AA vs GG + GA								
	overall	4827/6875	1.478 (0.821,2.662)	0.624	0.0%	0.193	0.161	0.034
	European	2108/3686	2.985 (1.121,7.946)	0.691	0.0%	0.209	0.624	0.902
	North Asian	2522/2785	0.947 (0.443,2.023)	0.659	0.0%	0.888	0.188	0.038
	HWE	3934/5941	2.832 (1.258,6.375)	0.903	0.0%	0.012	0.677	0.848
	NO HWE	696/530	0.602 (0.236,1.537)	0.721	0.0%	0.289	0.317	–
AA+GA vs GG								
	overall	4827/6875	1.072 (0.931,1.235)	0.033	46.6%	0.331	0.300	0.041
	European	2108/3686	0.929 (0.758,1.138)	0.375	5.6%	0.475	0.327	0.440
	North Asian	2522/2785	1.231 (1.010,1.500)	0.044	51.5%	0.040	0.621	0.148
	HWE	3934/5941	1.020 (0.879,1.184)	0.052	45.0%	0.791	0.186	0.110
	NO HWE	696/530	1.686 (1.060,2.681)	0.586	0.0%	0.027	0.317	–
AA vs GG								
	overall	4827/6875	1.506 (0.835,2.715)	0.658	0.0%	0.173	0.161	0.033
	European	2108/3686	2.961 (1.113,7.879)	0.691	0.0%	0.030	0.624	0.927
	North Asian	2522/2785	0.980 (0.458,2.097)	0.680	0.0%	0.958	0.188	0.037
	HWE	3934/5941	2.838 (1.260,6.394)	0.934	0.0%	0.012	0.677	0.893
	NO HWE	696/530	0.629 (0.246,1.608)	0.705	0.0%	0.333	0.317	–
GA vs GG								
	overall	4827/6875	1.165 (0.914,1.485)	0.007	56.2%	0.218	0.100	0.040
	European	2108/3686	0.890 (0.706,1.121)	0.343	11.1%	0.322	0.327	0.391
	North Asian	2522/2785	1.409 (0.981,2.024)	0.014	60.2%	0.063	0.805	0.160
	HWE	3934/5941	1.053 (0.837,1.325)	0.039	47.6%	0.659	0.186	0.156
	NO HWE	696/530	2.265 (1.307,3.926)	0.651	0.0%	0.004	0.317	–
A vs G								
	overall	4827/6875	1.088 (0.950,1.244)	0.096	35.8%	0.222	0.246	0.040
	European	2108/3686	0.979 (0.807,1.189)	0.416	0.0%	0.833	0.142	0.509
	North Asian	2522/2785	1.201 (0.995,1.450)	0.084	44.1%	0.057	0.805	0.117
	HWE	3934/5941	1.055 (0.914,1.217)	0.077	40.7%	0.465	0.243	0.087
	NO HWE	696/530	1.377 (0.918,2.065)	0.538	0.0%	0.122	0.317	–

^*^P value of Heterogeneity chi-squared

^#^P value of Pooled statistic

### Meta-analysis of *TNF-α* 857C/T polymorphisms and CAD susceptibility

In total, 9 studies with3843 patients in the CAD group and 5616 in the control group reported on the association of *TNF-α* 857C/T and CAD susceptibility. For studies with no significant heterogeneity (chi-squared test *P* > 0.05 and I^2^ < 50%), the fixed-effects model was chosen to analyze all the comparison models. The results of Begg’s test (*p* > 0.05) revealed no significant publication bias among the study results.

**Fig. 6:**
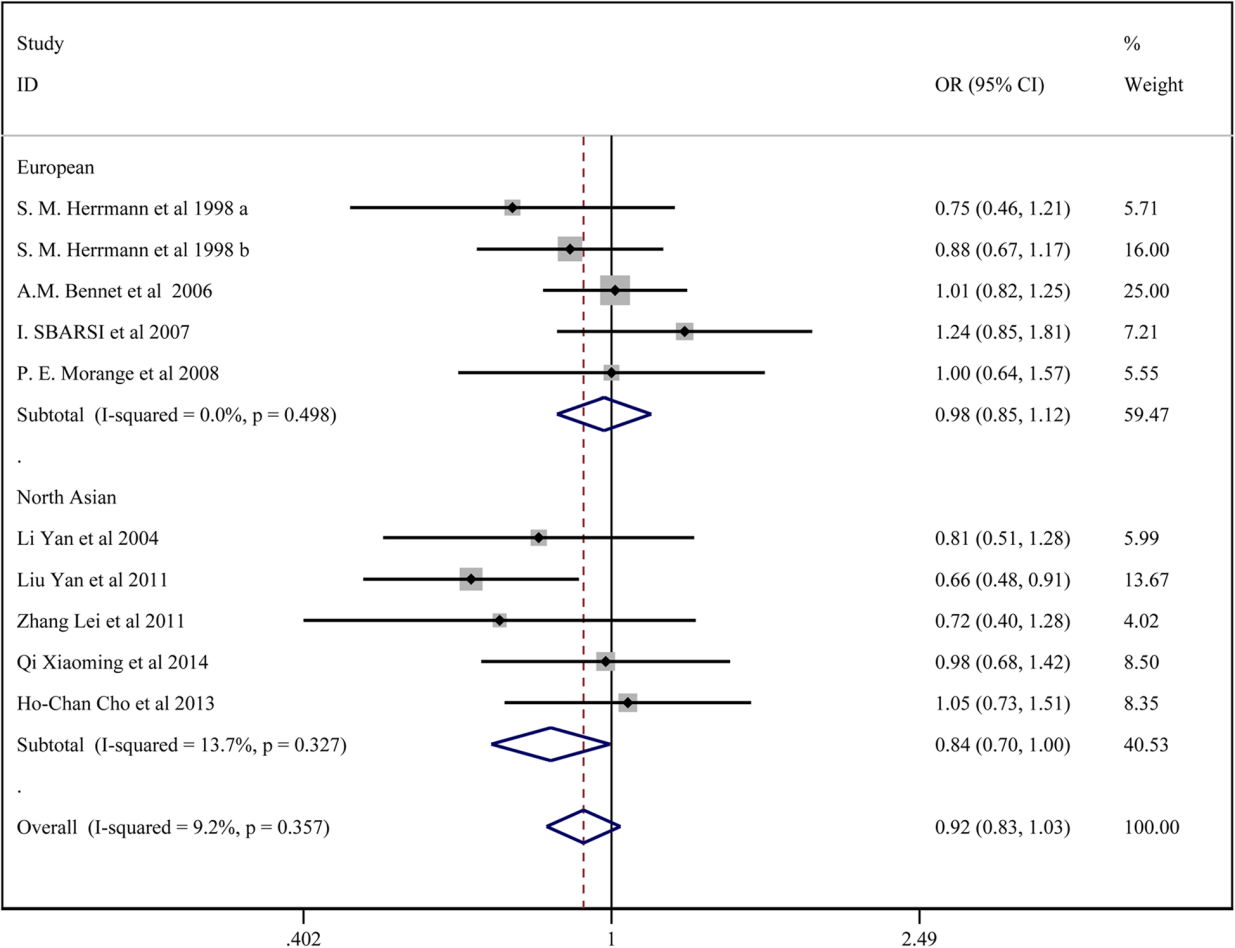
Forest plot for the dominant model of TNF-α 857C/T polymorphisms associated with CAD

The results showed no significant association between *TNF-α* 857C/T locus T and CAD susceptibility: the allelic model (T vs. C) (OR:0.949, 95% CI:0.862–1.045); the homozygote model (TT vs. CC) (OR:1.105, 95%CI:0.820–1.488); the dominant model (TT + CT vs. CC) (OR: 0.920, 95% CI:0.825–1.027); the regressive model (TTvs.CC+ CT) (OR: 1.124, 95% CI: 0.836–1.510); the heterozygote model (CT vs. CC) (OR: 0.904, 95% CI:0.807–1.012). In the subgroup analysis, there was no significant association between TNF-α 857C/T and CAD by the five models in Europeans, HWE and no HWE. In the north Asian population, *TNF-α* 857C/T locus T was associated with lower CAD susceptibility by the heterozygote model (CT vs. CC) (OR: 0.812, 95% CI:0.676–0.976), the dominant model (TT + CT vs. CC) (OR: 0.835, 95% CI:0.701–0.996);

All the above results are presented in Fig. 6 and Table 4.

**Table 4:** Meta-analysis of TNF-α 857C/T polymorphisms and CAD susceptibility

Genetic Model	Subgroup analysis	N (case/control)	OR(95%CI)	P*	I2	P#	P value	
							Begg	Egger
TT vs CC + CT								
	overall	3494/5279	1.124 (0.836,1.510)	0.437	0.0%	0.440	1.000	0.769
	European	2139/3566	1.135 (0.753,1.710)	0.752	0.0%	0.546	0.624	0.577
	North Asian	1158/1309	1.112 (0.726,1.703)	0.132	43.5%	0.627	0.624	0.994
	HWE	1844/3078	1.230 (0.866,1.748)	0.466	0.0%	0.247	0.881	0.960
	NO HWE	1453/1797	0.901 (0.519,1.564)	0.305	15.9%	0.711	0.602	0.839
TT + CT vs CC								
	overall	3494/5279	0.920 (0.825,1.027)	0.357	9.2%	0.137	0.283	0.467
	European	2139/3566	0.978 (0.851,1.125)	0.498	0.0%	0.758	0.327	0.810
	North Asian	1158/1309	0.835 (0.701,0.996)	0.327	13.7%	0.045	0.624	0.992
	HWE	1844/3078	0.909 (0.793,1.041)	0.230	26.1%	0.167	0.881	0.641
	NO HWE	1453/1797	0.942 (0.784,1.132)	0.428	0.0%	0.524	0.117	0.006
TT vs CC								
	overall	3494/5279	1.105 (0.820,1.488)	0.368	8.0%	0.513	0.858	0.833
	European	2139/3566	1.140 (0.755,1.721)	0.704	0.0%	0.534	0.624	0.643
	North Asian	1158/1309	1.067 (0.693,1.644)	0.109	47.1%	0.767	0.624	0.972
	HWE	1844/3078	1.209 (0.848,1.724)	0.383	5.8%	0.295	0.881	0.996
	NO HWE	1453/1797	0.890 (0.511,1.547)	0.291	19.1%	0.679	0.602	0.872
CT vs CC								
	overall	3494/5279	0.904 (0.807,1.012)	0.605	0.0%	0.081	0.474	0.429
	European	2139/3566	0.966 (0.836,1.116)	0.610	0.0%	0.637	0.327	0.689
	North Asian	1158/1309	0.812 (0.676,0.976)	0.649	0.0%	0.026	1.000	0.695
	HWE	1844/3078	0.881 (0.765,1.015)	0.466	0.0%	0.080	0.652	0.632
	NO HWE	1453/1797	0.946 (0.782,1.145)	0.513	0.0%	0.569	0.602	0.247
T vs C								
	overall	3494/5279	0.949 (0.862,1.045)	0.181	28.6%	0.288	0.371	0.496
	European	2139/3566	0.994 (0.878,1.126)	0.442	0.0%	0.926	0.624	0.901
	North Asian	1158/1309	0.886 (0.762,1.032)	0.108	47.2%	0.119	1.000	0.718
	HWE	1844/3078	0.952 (0.846,1.071)	0.109	42.2%	0.416	0.652	0.796
	NO HWE	1453/1797	0.943 (0.798,1.114)	0.330	9.7%	0.490	0.117	0.223

*P value of Heterogeneity chi-squared

#P value of Pooled statistic

### Meta-analysis of *TNF-α* 863C/a polymorphisms and CAD susceptibility

In total, 10 studies with3225 patients in the CAD group and 4784 controls reported on the association of TNF-α 863C/A and CAD susceptibility. For studies with no significant heterogeneity (chi-squared test, *P* > 0.05 and I^2^ < 50%), the fixed-effects model was chosen to analyze the regressive model and homozygote model, while other models were analyzed using the random-effects model. The results of Begg’s test (*p* > 0.05) showed no significant publication bias in the results of the regressive model and homozygote model.

The results showed no significant association between *TNF-α* 863C/A locus A and CAD susceptibility: the allelic model (A vs. C) (OR:0.803, 95% CI:0.584–1.103); the homozygote model (AA vs. CC) (OR:0.838, 95% CI:0.612–1.145); the dominant model (AA + CA vs. CC) (OR: 0.793, 95% CI:0.512–1.227); the regressive model (AA vs.CA + CC) (OR:0.828, 95% CI: 0.608–1.129); the heterozygote model (CA vs. CC) (OR: 0.805, 95% CI:0.584–1.103).

**Fig. 7:**
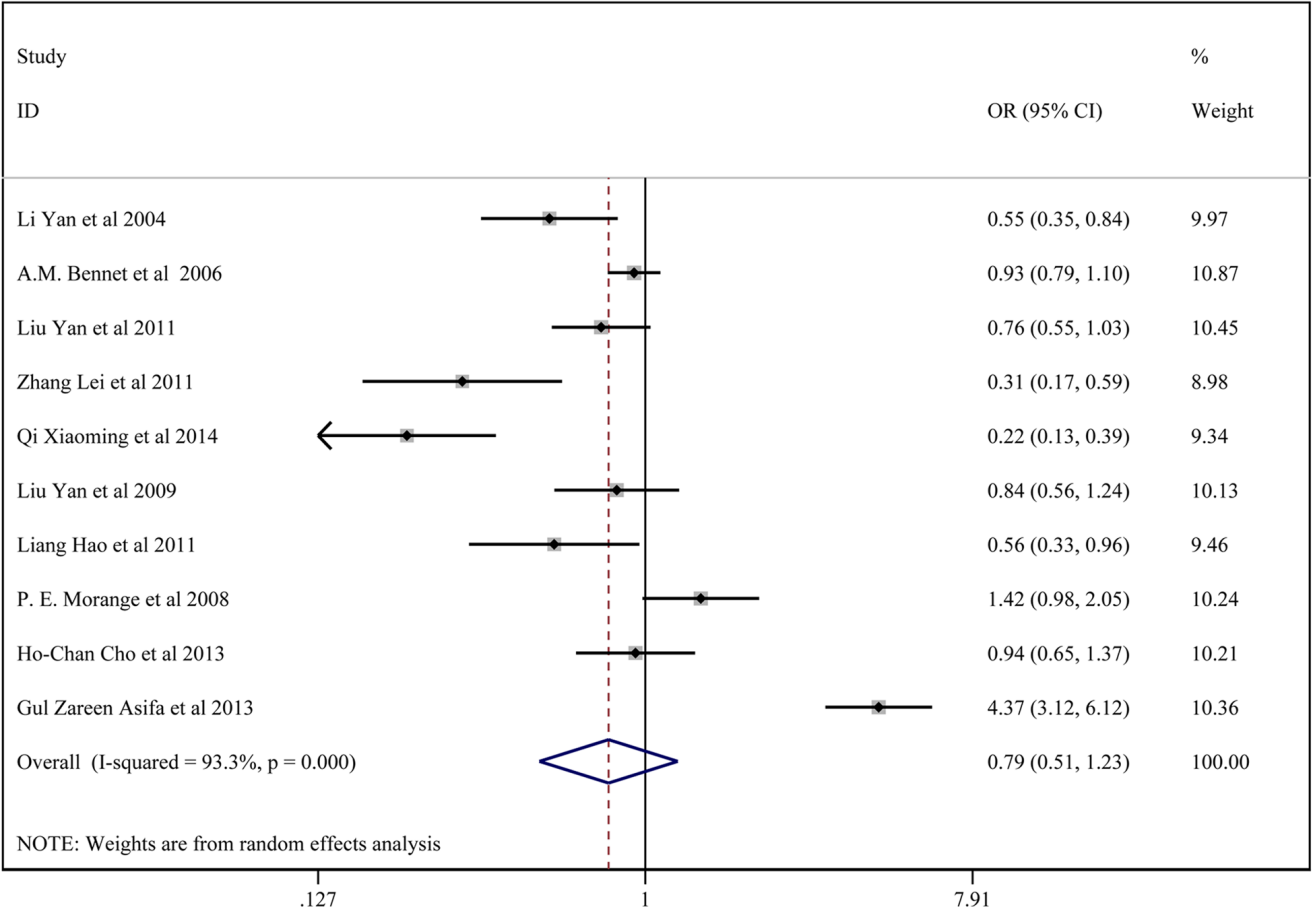
Forest plot for the dominant model of TNF-α 863C/A polymorphisms associated with CAD

All the above results are presented in Fig. 7 and Table 5.

**Table 5:** Meta-analysis of TNF-α 863C/A polymorphisms and CAD susceptibility

Genetic Model	N (case/control)	OR(95%CI)	P*	I2	P#	*P* value	
						Begg	Egger
AA vs CC + CA	3144/4491	0.828 (0.608,1.129)	0.478	0.0%	0.234	0.466	0.016
AA+CA vs CC	3144/4491	0.793 (0.512,1.227)	0.000	93.3%	0.298	0.020	0.390
AA vs CC	3144/4491	0.838 (0.612,1.145)	0.450	0.0%	0.267	0.348	0.035
CA vs CC	3144/4491	0.805 (0.513,1.265)	0.000	93.3%	0.347	0.032	0.426
A vs C	3144/4491	0.803 (0.584,1.103)	0.000	90.6%	0.176	0.012	0.204

*P value of Heterogeneity chi-squared

#P value of Pooled statistic

### Meta-analysis of *TNF-α* 1031 T/C polymorphisms and CAD susceptibility

In total, 9 studies with 3851 patients in the CAD group and 3936 controls reported on the association between *TNF-α* 1031 T/C and CAD susceptibility.

For studies with no significant heterogeneity (chi-squared test, *P* > 0.05 and I^2^ < 50%), the fixed-effects model was chosen to analyze all the comparison model except the regressive model and homozygote model. The results of Begg’s test (*p* > 0.05) showed no significant publication bias among the study results.

The results showed no significant association between *TNF-α* 1031 T/C locus C and CAD susceptibility: the allelic model (C vs. T) (OR:0.973, 95% CI:0.898–1.054); the homozygote model (CC vs. TT) (OR:0.999, 95% CI:0.666–1.498); the dominant model (CC + CT vs. TT) (OR: 0.945, 95% CI:0.860–1.039); regressive model (CC*vs.*TT+ CT) (OR: 1.020, 95% CI: 0.677–1.539); the heterozygote model (CT *vs.* TT) (OR: 0.929, 95% CI:0.842–1.025).

**Fig. 8:**
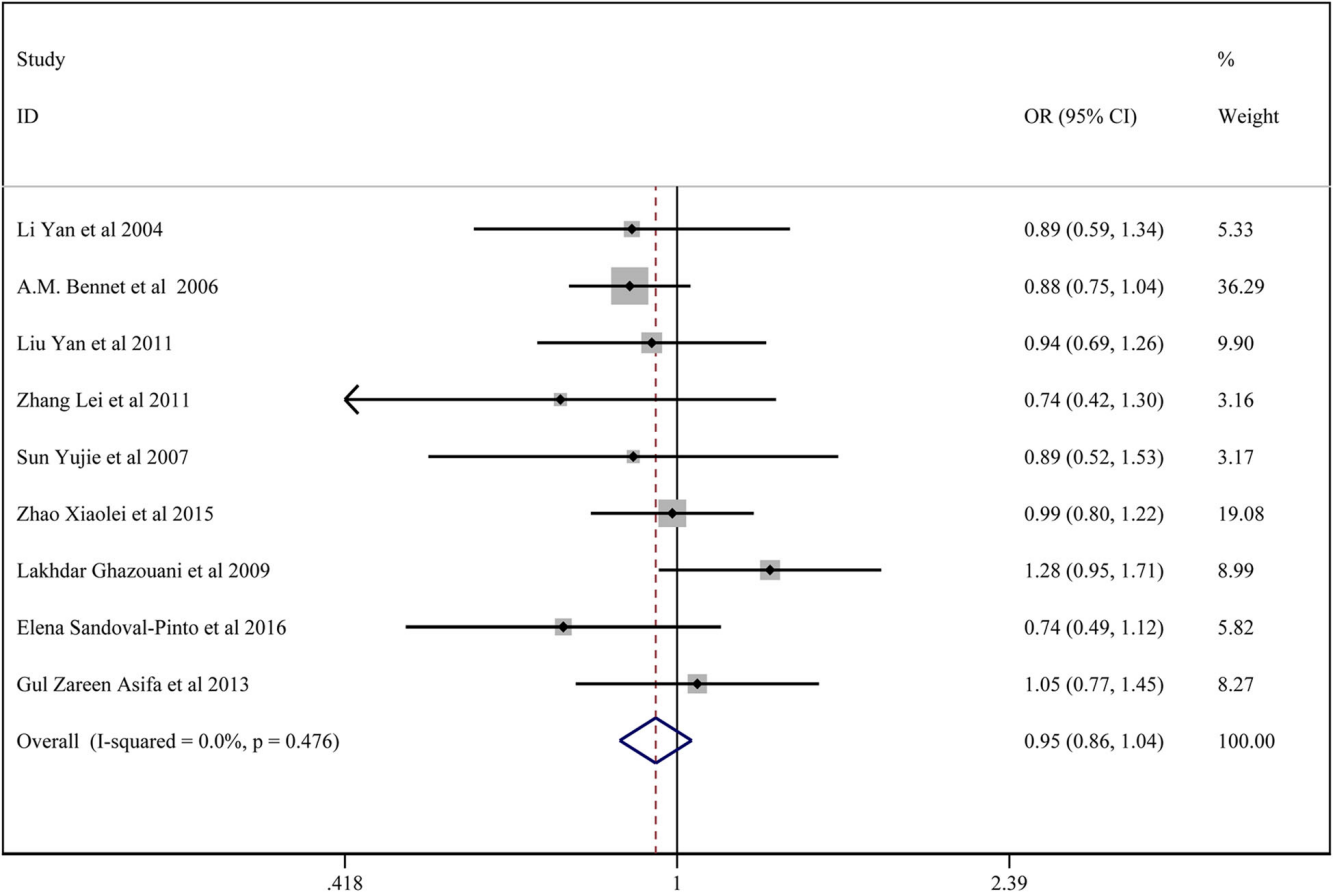
Forest plot for the dominant model of TNF-α 1031 T/C polymorphisms associated with CAD

All the above results are presented in Fig. 8 and Table 6.

**Table 6:** Meta-analysis of TNF-α 1031 T/C polymorphisms and CAD susceptibility

Genetic Model	N (case/control)	OR(95%CI)	P*	I2	P#	*P* value	
						Begg	Egger
CC vs TT + CT	3781/3845	1.020 (0.677,1.539)	0.013	58.6%	0.923	0.602	0.458
CC + CT vs TT	3781/3845	0.945 (0.860,1.039)	0.476	0.0%	0.243	0.466	0.786
CC vs TT	3781/3845	0.999 (0.666,1.498)	0.018	56.5%	0.997	0.602	0.465
CT vs TT	3781/3845	0.929 (0.842,1.025)	0.401	4.1%	0.141	0.175	0.951
C vs T	3781/3845	0.973 (0.898,1.054)	0.248	21.9%	0.505	1.000	0.624

*P value of Heterogeneity chi-squared

#P value of Pooled statistic

## Discussion

Atherosclerosis is the pathological basis of coronary heart disease, and inflammation plays a crucial role in the occurrence and development of atherosclerosis. Inflammation plays an important role in the formation, growth, rupture, and/or wear and tear of atherosclerotic plaques and the formation of blood clots. In particular, acute cardiovascular events such as heart failure, nausea and arrhythmia, cardiogenic shock and even cardiac arrest caused by plaque rupture and secondary acute thrombosis leading to complete occlusion of blood vessels are common clinical emergencies with sudden onset and high mortality. Therefore, the occurrence and development of coronary heart disease is a process of chronic inflammatory response.

TNF-α is an important proinflammatory cytokine mediating inflammatory response and immune regulatory response in vivo. TNF-α can affect the development of coronary heart disease through the following ways: [1] participation in the inflammatory response of atherosclerotic plaques, the formation and rupture of plaques, leading to coronary heart disease and even acute myocardial infarction [2]. Direct injury to vascular endothelial cells can increase their permeability, and blood cholesterol can easily penetrate the intima and deposit in the wall of the vessels [3]. Promotion of proto-oncogene transcription, production of platelet-derived growth factors, disruption of the balance between blood coagulation and anti-blood coagulation, and promotion of thrombosis [4]. Inhibiting lipoprotein enzyme activity is not conducive to lipid dissolution and deposition in the vascular wall, promoting the formation of arteriosclerosis and aggravating the damage of the vascular wall. *TNF-α* polymorphic loci are located in the promoter region of − 308, − 238, − 163, − 244,-857, − 836, − 1031 and other loci. The presence of these gene polymorphisms may affect gene transcription and expression levels and be associated with various diseases.

In previous studies, Fengtian et al. [35] included 14 studies and found no association between T-1031C, C-857 T and C-863A and CAD risk. Karely et al. [36] included 27 articles, and found a significant association between *TNF-a* G308A and CHD in the whole population, and between the variant G238A and CHD in the Asian population.

In our study, we found that *TNF-α* 308G/A locus A had no significant association with CAD susceptibility by the five models in the analysis of the overall population, Europeans, Africans, south Asians, and north Asians, which is contrary to the conclusion of Karely Pulido-Gomez. *TNF-α* 863C/A locus A and 1031 T/C locus C showed no significant association with CAD susceptibility, which is consistent with the conclusion of Fengtian HUANGFU. *TNF-α* 238G/A locus A had no significant association with CAD susceptibility in the overall population. However, TNF-α 238G/A locus A displayed significant association with higher CAD susceptibility in the subgroup of Europeans and north Asians. The association of TNF-α 238G/A in Asians is consistent with the study by Karely Pulido-Gomez. *TNF-α* 857C/T locus T had no significant association with CAD susceptibility in the analysis of the overall population and Europeans. In the north Asian population, *TNF-α* 857C/T locus T was associated with lower CAD susceptibility.

However, there are certain limitations to the present analysis, which are as follows: [1] only English and Chinese articles were included [2]; individual studies had different exclusion/inclusion criteria [3]; the severity of CAD was varied in different studies [4]; the number of included studies was limited, and some of the studies had a small sample size [5]; pooled data were analyzed, as individual patient data was not available, precluding more in-depth analyses.

## Conclusion

Our results indicate that *TNF-α* 308G/A, 857C/T, 863C/A, and 1031 T/C are not associated with CAD susceptibility. *TNF-α* 238G/A locus A has significant association with CAD susceptibility In Europeans and north Asians, but has no significant association in the overall population. In the north Asian population, *TNF-α* 857C/T locus T was associated with lower CAD susceptibility. Larger-sample studies are required to confirm the association between *TNF-α* 238G/A and 857C/T and CAD susceptibility.

## Data Availability

All data generated or analysed during this study are included in this published article.

## Abbreviations

ACS: Acute coronary syndrome
AS: Atherosclerosis
CAD: Coronary artery disease
HWE: Hardy-Weinberg equilibrium
NOS: Newcastle–Ottawa Scale
PICOS: Participants, Interventions, Comparisons, Outcomes and Study design
TNF: Tumor necrosis factor
TNF-α: Tumor necrosis factor-α
